# Thematic and intertextual analysis from a feasibility study of the Bonny Method of Guided Imagery and Music with clients in eating disorder treatment

**DOI:** 10.3389/fpsyg.2024.1456033

**Published:** 2024-10-31

**Authors:** Annie Heiderscheit

**Affiliations:** Cambridge Institute for Music Therapy Research, Faculty of Arts, Humanities, Education and Social Sciences, Creative Industries, Anglia Ruskin University, Cambridge, United Kingdom

**Keywords:** GIM, music therapy, eating disorders, imagery themes, neuroaesthetics

## Abstract

**Introduction:**

Eating disorders (ED) are characterized by serious and persistent disturbances with eating, weightcontrol, and body image. Symptoms impact physical health, psychosocial functioning, and can be life-threatening. Individuals diagnosed with an ED experience numerous medical and psychiatric comorbidities due to issues caused by or underlying the ED. Therefore, it is vital to address the complex nature of an ED, as well as the comorbid and underlying issues. This necessitates a psychotherapeutic approach that can help to uncover, explore, and support working through unresolved emotions and experiences. Guided Imagery and Music (GIM) is an in-depth music psychotherapy approach utilizing therapist-programmed music to support the client in uncovering and examining underlying and unresolved issues. The literature surrounding the use of GIM with clients in ED treatment is anecdotal and comprised primarily of clinical case studies.

**Method:**

This secondary analysis, based on a descriptive feasibility study that integrated GIM sessions into the client’s regular ED treatment and examined 116 transcripts from a series of sessions of eight clients.

**Results:**

Thematic analysis of the transcripts identified nine subthemes and three themes that emerged. These themes include emotional landscape (feeling stuck, acknowledging emotions, and working through unresolved emotions), relationships (self, others, and eating disorders), and transformation and growth (finding strength, change, and empowerment). A short series of GIM sessions helped ED clients identify and address issues underlying the ED and to gain or reclaim a sense of self that enabled them to make choices for their life that support their recovery and sense of empowerment. Intertextual analysis revealed imagery indicative of the *Hero’s Journey*.

**Discussion:**

Further, how engagement in this embodied aesthetic experience stimulates perceptual, cognitive, and affective brain functions which are key in fostering behavioural and psychological change is explicated as it relates to ED treatment and recovery.

## 1 Background

The hallmark characteristics of eating disorders (EDs) include the persistent disturbance of eating behaviors, weight control, and body image. These behaviors significantly impact one’s relationship with their body and weight, as well as their physical health and psychosocial functioning (American Psychological Association, 2013). Accurate estimates regarding how many individuals worldwide struggle with an ED are challenging, as many do not seek treatment due to feeling embarrassed to report symptoms or being in denial of symptoms (Smink et al., 2012; Steward and Robinson, 2018). Projections indicate that 70 million people worldwide are diagnosed with an ED and incidence rates have been increasing since early 2000, including increases in severe ED cases and a 66% increase in hospital admissions in 2020 (Beat Eating Disorders, 2021; Hott et al., 2015; Keel, 2018; Keski-Rahkonen et al., 2007; Rikani et al., 2013; Steinhausen and Jensen, 2015; STRIPED Harvard, 2020).

While EDs are characterized by a range of behaviors related to eating, weight, and body image, these manifest differently with each diagnosis. Individuals diagnosed with anorexia nervosa (AN) experience low body weight and an intense fear of gaining weight, while those with Bulimia nervosa (BN) experience episodes of bingeing and purging. Individuals with binge eating disorders (BED) demonstrate a lack of control related to eating and engage in overeating food in a short period of time (American Psychological Association, 2013). EDs are not characterized by a single risk factor or cause. The growing body of literature indicates there are multiple risk factors that impact the development of an ED (Ely and Kaye, 2018). These include three distinctive areas: (1) genetic, (2) psycho-developmental, and (3) sociocultural factors (Anderson-Fye, 2018; Jacobi et al., 2018; Stice and Shaw, 2018; Vögele et al., 2018; Wade and Bulik, 2018; Wade et al., 2006). Researchers and clinicians recognize that the entangled nature of these risk factors along with comorbid health issues and mental health diagnoses can prolong ED symptoms (Rikani et al., 2013). This type of complex constellation suggests that attention be given to understanding if a risk factor is an integral part of the ED (i.e., body dissatisfaction) or the effect of the prolonged disordered eating or symptom use (i.e., mood disturbances or changes in serotonin) (Abbate-Daga et al., 2014; Backholm et al., 2013; Biffl et al., 2010; Braun et al., 1994; Bulik et al., 2004; Costello and Angold, 1995; Faje et al., 2014; Fairburn et al., 1999; Halmi et al., 2000; Wade and Bulik, 2018). This broaden perspective recognizes that one’s overall health is impacted by contextual and interactive factors, as well as an understanding of the unique characteristic of their illness (Martin and Felix-Bortolotti, 2010).

## 2 Eating disorders: a complex clinical profile

The concept of a complex clinical profile is a broad way of understanding that health and various aspects that contribute to one’s health are impacted and complicated by one’s unique circumstances (Manning and Gagnon, 2017; Martin and Felix-Bortolotti, 2010). In the context of an individual diagnosed with an ED, psychological and medical comorbidities, as well as social and cultural factors impact the treatment and recovery process (Anderson-Fye, 2018; Halmi, 2018; Mehler, 2018). This acknowledges the interactive nature of these comorbidities and sociocultural factors and their collective impact on ED treatment and recovery.

Individuals diagnosed with an ED experience a significant incidence of psychiatric comorbidities, specifically anxiety disorders, substance use disorders, personality disorders, and post-traumatic stress disorder (PTSD) (Braun et al., 1994; Costello and Angold, 1995; Faje et al., 2014; Halmi et al., 2000; Herzog et al., 1992; Hudson et al., 2007; Kaye et al., 2004; Tagay et al., 2014). Medical complications are common due to weight loss, malnutrition, purging, and overeating (Mehler, 2018). The frequency and severity of ED behaviors impacts every system of the body causing cardiac complications, pulmonary abnormalities, gastrointestinal issues, hematologic disorders (i.e., leukopenia and anemia), diminished neurological functioning, impaired endocrine processing, dermatological issues (i.e., thinning hair, brittle nails, cyanotic extremities, and lanugo hair growth on the face), and reduced bone metabolism (leading to osteopenia and osteoporosis) (Biffl et al., 2010; Faje et al., 2014; Kaplan and Nobel, 2018; Kraeft et al., 2013; Lamzabi et al., 2015; Mascolo et al., 2015; Mehler, 2018; Miller et al., 2004; Roberto et al., 2011; Sabel et al., 2013; Strumia, 2012; Westermoreland et al., 2016). There are also personality traits (i.e., perfectionism) that are intertwined with and impact the ED (Burke et al., 2018; Chen et al., 2018; Keel and Brown, 2010; LaMarre and Rice, 2021; Le Grange and Rienecke, 2018; Lester, 2019; Silber et al., 2011; Steward and Robinson, 2018; Wonderlich et al., 2012; Zander and Young, 2014).

This complex collection of symptoms also impacts an individual’s thought patterns, affective processing, and mood states and while these can be common issues to address, ED diagnoses differ regarding their symptomatologic constellation and clinical presentation. A growing body of literature indicates that neural activity and neurofunctional correlates distinct to each disorder occur in different areas of the brain (Celeghin et al., 2023; Wonderlich et al., 2021). Individuals with AN demonstrate a hyperactivity in the amygdala (AMG), insula (INS), and hypothalamus that is associated with control and emotion dysregulation (Carlèn, 2017; Celeghin et al., 2023; Wonderlich et al., 2021). Clients with BN struggle with impulsivity and emotion regulation, which is a result of hyperactivity in the INS and striatum (STR); whereas individuals with BED display dissociative and addictive behaviors which are indicated in the temporal cortex and STR (Celeghin et al., 2023; Wonderlich et al., 2021).

Individuals diagnosed with EDs also report a high incidence of childhood maltreatment and having experienced traumatic events (Brewerton, 2019). Addressing these diverse and myriad needs warrants treating the client wholistically by understanding their clinical manifestation, recognizing that EDs develop through a multi-causal process, and that the interaction of these different factors necessitate tailoring treatment (Hester and Sheehy, 1990; Heiderscheit, 2008; Le Grange and Eisler, 2009; Linehan, 2014; Le Lock et al., 2001; McFerran and Heiderscheit, 2015; McNeece and DiNitto, 2011; Trondalen, 2016b; Celeghin et al., 2023). Further, the psychotherapeutic therapeutic process requires structure and pacing to foster safety to avoid triggering ED behaviors or trauma (Burke et al., 2018; Dyer and Corrigan, 2021; Favaro et al., 2003; Guarda et al., 2018; Heiderscheit and Madson, 2015; Heiderscheit and Murphy, 2021; Ogden et al., 2006; van der Kolk, 2014; Wilson, 2018).

### 2.1 Eating disorder treatment

There are several therapeutic approaches commonly implemented in ED treatment which include cognitive behavioral therapy (CBT), dialectical behavior therapy (DBT), family-based therapy (FBT) and interpersonal therapy (IPT) (Agras and Robinson, 2018). Each therapeutic approach has a unique focus, with CBT targeting thoughts and feelings related dysfunctional evaluation of one’s weight and body, DBT aimed at addressing affect regulation, FBT utilizing the family as a resource to reduce and eliminate ED behaviors, and IPT striving to develop awareness of how interpersonal relationships impact the ED (Burke et al., 2018; Chen et al., 2018; Le Grange and Rienecke, 2018; Wilson, 2018). While each approach addresses different therapeutic needs, research indicates ED treatment is effective for 50% of clients (Levinson et al., 2021).

There is limited research of the use of creative arts therapies (CATs) in ED treatment (Heiderscheit, 2015a), yet emerging evidence the field of neuroaesthetics indicates that the aesthetic experience is a necessary and instrumental, as it stimulates perceptual, cognitive, and affective brain functions that are key in fostering behavioral and psychological change (Vaisvaser et al., 2024). It is postulated that the complex interplay of therapeutic factors in the aesthetic experience, create space for the client’s context and subjective experiences to be key elements in making meaning and fostering change in the therapeutic process (Jacobsen, 2006; Vaisvaser et al., 2024). The aesthetic experience fosters clients experience of therapeutic factors including externalization, embodiment, and symbolization (Kushnir and Orkibi, 2021; Kirsch et al., 2016; de Witte et al., 2021).

In the CATs, the externalization process allows an individual to cognitively shape the therapeutic issue in a manner that provides a sense of safety and to exert control over their engagement with it. Their ability to create this distance allows them to feel the intensity of the emotions, to be immersed in the moment and to feel safe (Vaisvaser et al., 2024). Processing these emotions fosters embodiment which facilitates the brain–body–mind connection, changing one’s perception and awareness as they access body memories (Vaisvaser, 2021; Vaisvaser et al., 2024). Lastly, in CATs, symbolic and metaphoric representations of the client’s inner experiences are expressed by bringing the subconscious into conscious awareness, thus offering opportunity for meaning making (Imus, 2021; Vaisvaser et al., 2024).

Aesthetic experiences that are inherent in CATs support increased connectivity and activity in various neural networks, including motor, reward, and sensory networks, as well as salience network, which is integral to interoceptive inference and awareness (Kennett et al., 2023; Vaisvaser et al., 2024). Engaging multisensory experiences fosters an integration of interoceptive, proprioceptive, and exteroceptive input that supports as a client’s sense of agency, as they can direct and control the ways they engage (Salvato et al., 2019). One such CATs method that provides this aesthetic experience and fosters the brain–body–mind connection is the Bonny Method of Guided Imagery and Music (GIM).

### 2.2 GIM in ED treatment

GIM is an in-depth music psychotherapy approach integrating listening to sequenced classical music to elicit and explore feelings, images, experiences, and memories. GIM helps to foster insights, develop awareness and self-understanding, as well as support personal growth and development (Bonny, 1994; Bonny and Savary, 1990; Grocke, 2019). This receptive method utilizes therapist-selected and programmed music specifically designed to meet the individual where they are in their therapeutic process and adapted to meet their needs moment to moment (Heiderscheit, 2023). GIM is designed to uncover unexpressed emotions and unresolved issues from the subconscious to allow the client to bring them into their conscious awareness (Ahonen, 2019; Beck et al., 2018; Bonny, 1994; Bonny and Savary, 1990; Clark and Keiser, 1986; Grocke, 2019; Goldberg, 1995; Goldberg, 2019; Heiderscheit, 2017). As clients listen to music in a relaxed state, they can access unresolved and repressed thoughts, feelings, and experiences, projecting them onto the music and imagery to bring them into their conscious awareness for exploration and discovery (Pickett, 1991).

The literature surrounding the use of GIM in ED treatment is limited to anecdotal evidence and clinical cases. This body of literature indicates that GIM has assisted clients in exploring and addressing unresolved feelings, confronting perfectionistic tendencies, working through body image issues, address anxiety, depression, and self-harm, work through trauma, explore origins of the ED, and discover inner resources (Barmore, 2017; Gargaro et al., 2016; Heiderscheit, 2016a, 2016b; Heiderscheit, 2015b; Heiderscheit, 2013; Noer, 2015; Papanikolaou, 2015; Pickett, 1991; Trondalen, 2016a; Trondalen, 2011). This clinical and anecdotal evidence indicates a need for more rigorous quantitative and qualitative research.

**Figure 1:**
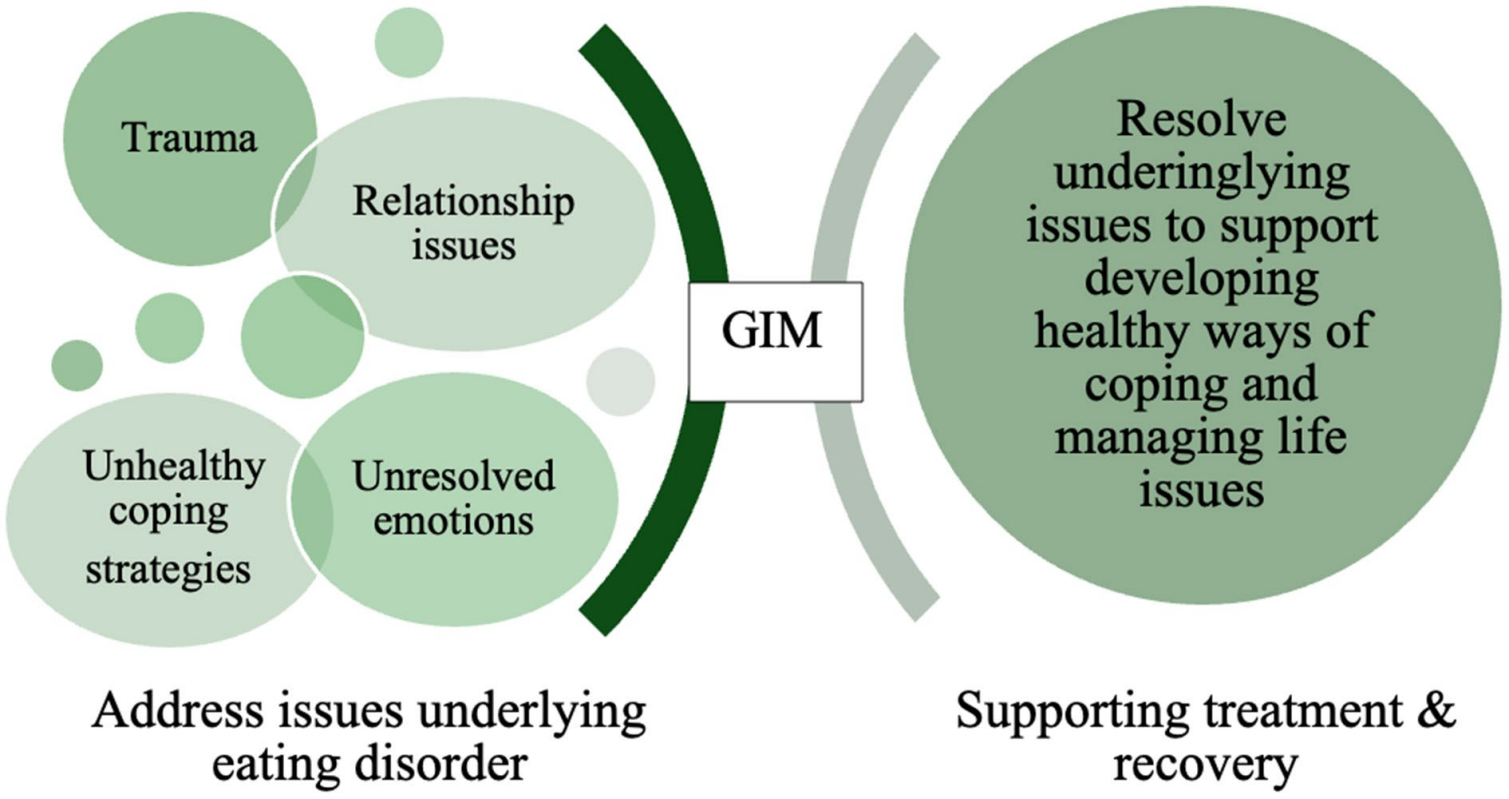
Conceptual framework.

This study is a secondary analysis of a parent study that was the first feasibility study that explored the use of GIM with individuals in ED treatment. The parent study was based on the conceptual framework included in Figure 1, that focuses on addressing issues underlying the ED to support the client in identifying and addressing these issues through a music psychotherapy method (GIM), designed to bring these unresolved issues/emotions into their conscious awareness. Developing awareness of these issues allows individuals access to explore and work through these issues, which helps to support their treatment process and recovery (Heiderscheit, 2017). Primary results from the parent study included participants reporting perceived benefits of engaging in a series of GIM sessions, in which they indicated GIM helped them develop insights related to feeling stuck, their fears, and needing to change. They also reported exploring emotional processes in GIM including identifying, experiencing, and processing their emotions. Additionally, they reported experiencing growth, which included discovering their inner strength, developing a belief in self, and learning to let go of their ED (Heiderscheit, 2023). While participant’s identified perceived benefits of GIM is valuable information for researchers and clinicians, it is also helpful to understand this in tandem with data that explores their therapeutic experiences via analysis of their music and imagery transcripts.

## 3 Methods

This secondary analysis was based on a descriptive feasibility parent study that explored the integration GIM in ED treatment. The aim of this secondary analysis was to understand participant’s experiences in GIM by identifying imagery themes and exploring the contextual nature of their therapeutic process. The aim for phase one (thematic analysis) was to understand the therapeutic issues that clients address in GIM, while the aim for phase two (intertextual analysis) was to understand these issues within the context of their therapeutic process and ED treatment. Thematic and intertextual methods of analysis were utilized to examine participant’s music and imagery experiences, how they describe their experiences, and to understand these experiences in the context of ED treatment. To explore participant’s experiences, transcripts from each GIM session were analyzed. All GIM sessions were facilitated by a board-certified music therapist (MT-BC), who is also licensed as a marriage and family therapist (LMFT) and certified in the Bonny Method of Guided Imagery and Music (GIM fellow).

### 3.1 Setting and participants

Participants in this study included clients engaged in an ED treatment program in a Midwest metropolitan area. Eligible clients were invited to participate in the study if they were actively engaged in ED treatment (including residential, intensive day treatment, intensive outpatient, and outpatient) and their treatment team determined that GIM was an appropriate therapeutic modality for their treatment process.

Approval for the study was obtained through the Institutional Review Board (IRB) at the University of Minnesota. Exclusion criteria included: (a) non-English speaking, (b) medically unstable, and (c) GIM was contraindicated due to dissociative tendencies or dissociation. Informational flyers were posted at the ED treatment program and clients interested in participating in the study needed to obtain approval from their primary therapist to confirm that the treatment team deemed GIM appropriate. After this confirmation, the research assistant met with participants to complete informed consent which specified written consent for the use of quotes from session transcripts and to publish study outcomes. Following the completion of the informed consent, the research assistant scheduled them for their initial session with the MT-BC. Participants continued their regular, which included transitioning to different levels of care when appropriate and necessary. Eight clients were approached to participate in the study, completed informed consent, and received a series of GIM sessions during their ED treatment.

Study participants included eight individuals who identified as female and were between 23 and 58 years of age and a mean age of 35.5 years. Participants had been living with their EDs between 2 and 32 years and for an average of 15 years. They had experienced between 1 and 15 previous ED treatment episodes, with a mean of 8 overall episodes. Their ED diagnoses include AN-Binge Purge (*n* = 2) Type, AN-Restrictive Type (*n* = 3), OSFD-BN (*n* = 1), and BN (*n* = 1) and BED (*n* = 1). Table 1 provides participant demographic data including gender, ethnicity, age, ED diagnosis, comorbid mental health diagnoses, and number of previous ED treatment episodes. Participants were also diagnosed with multiple co-morbid mental health diagnoses: 86% with anxiety disorder, 75% with major depression, 63% with post-traumatic stress disorder, 38% with obsessive-compulsive disorder, 25% with borderline personality disorder, and 25% with bipolar disorder.

**Table 1:** Participant demographic.

Ethnicity and gender	Age	ED diagnosis	Comorbid mental health diagnoses	# of previous ED treatment episodes
White and F	27	Anorexia Nervosa-BP	Anxiety Disorder Major Depression Obsessive Compulsive Disorder Post-traumatic stress disorder	7
White and F	25	Other Specified Eating Disorder-Bulimia nervosa of low frequency	Anxiety Disorder Major Depression Post-traumatic stress disorder	8
White and F	23	Anorexia Nervosa-R	Anxiety Disorder Borderline Personality Disorder Post-traumatic stress disorder	5
White and F	28	Anorexia Nervosa-R	Anxiety Disorder Major Depression Bipolar Disorder Obsessive Compulsive Disorder	6
White and F	22	Anorexia Nervosa-R	Anxiety Disorder Obsessive Compulsive Disorder	1
White and F	35	Anorexia Nervosa-BP	Anxiety Disorder Major Depression Post-traumatic stress disorder	8
White and F	29	Bulimia Nervosa	Anxiety Disorder Major Depression Bipolar Disorder	9
White and F	58	Binge Eating Disorder	Major Depression Personality Disorder Post-traumatic stress disorder	13

### 3.2 Materials

GIM sessions occurred over a 12-month period as participants engaged in ED treatment and transitioned through levels of care. The frequency of GIM sessions was determined based on individual therapeutic needs and pacing appropriate for each participant. This resulted in sessions occurring either weekly or every other week. Sessions were tailored to accommodate a 50-min therapeutic hour due to the schedule and structure of the treatment program. Tailoring sessions for the therapeutic hour required shortening the music portion of sessions when longer programs were selected for use (Heiderscheit, 2015b). Treatment fidelity was maintained by including the following components in each GIM session and tracking these on session transcripts: 10–12 min of check-in and prelude, 25–30 min of music and imaging, and a 10–12-min postlude (Heiderscheit, 2023). All sessions were conducted in a private therapy office with a sofa and recliner to allow participants to choose whether they wanted to lay down or sit in a reclining position during sessions. Pillows and blankets were also available for participants’ comfort. The music utilized was the Music for Imagination^®^ (Bruscia, 1998) and was played from an Apple iPad Mini™ and through a Bose™ Bluetooth speaker.

Each session began with a check-in to allow the MT-BC to gather information, provide an update, and understand how the participant was doing, as well as to be updated about any changes in their life, and discuss any questions or insights from the previous session. This check-in allowed the MT-BC to assess the participant’s needs, including new developments in their treatment process, mood, energy level, as well as insights from previous session(s). This information was integrated with the GIM therapist’s knowledge of the individual’s previous session work to inform clinical decisions about the selection of music to tailor the session. Once the music was selected the participant was invited to lay down or move to a reclining position. The MT-BC then guided the participant through a brief relaxation experience (3–5 min) to shift their attention away from external and distracting thoughts and begin to focus inward. Then a starting image was introduced, which was informed by what the participant shared during the check-in discussion, as well as by their therapeutic work in the previous session. After the brief relaxation experience, music listening began. As the participant listened to the music, they are described aloud what they were imagining. Imagery includes what the participant was seeing, hearing, feeling, sensing, and experiencing. Throughout the process, the MT-BC asked questions to clarify and deepen the participant’s music and imagery experience. As the participant described their experiences, the MT-BC wrote down in a session transcript what they said, as well as the questions asked throughout the process. When the music ended the MT-BC directed the participant to bring their image to a close and guided them to focus their awareness on their breathing and the space around them. Following the music and imagery experience, the MT-BC engaged participants in a discussion identifying significant moments, sharing their feelings and any insights from their experience.

Participants were provided a copy of each session transcript, and the MT-BC maintained a transcript of each session as well. Participants were engaged in their current treatment episode between 12 and 40 weeks for a mean of 25.5 weeks when they began GIM sessions and transitioned to different levels of care as a part of their overall treatment process when necessary. Participants received between 11 and 17 GIM sessions with a mean of 14.5, which resulted in 116 GIM sessions overall. Throughout the study some sessions needed to be rescheduled, and a few sessions were missed due to outside appointments, work or family commitments, illness, and treatment programming changes or outings.

### 3.3 Data analysis

The review of 116 GIM transcripts was completed independently by two reviewers, including the MT-BC/researcher and an outside reviewer who was an experienced GIM therapist. Duplicates of the session transcripts were provided to the outside reviewer to complete their independent review. Phase one utilized a reflexive thematic analysis approach to embrace the GIM therapist/researcher’s subjectivity as a resource to understanding the client and their therapeutic process (Braun and Clarke, 2023a). This approach acknowledges the interpretive nature of the analysis, so to work on keeping researcher bias in check, coding reliability was implemented with the outside reviewer. The reflexive analysis process included reviewing and coding of all transcripts manually following the six-step process (Braun and Clarke, 2023a, 2023b). Emergent themes were identified based on clients’ words and descriptions of their music and imagery experiences, as well as any explanatory comments they made during prelude and postlude discussions. The inclusion of the prelude and postlude dialogue and comments on the transcripts helped to ensure the themes reflected and remained close to participant’s description of their experiences and informed reviewers’ understanding. This iterative process included clustering and grouping these themes into categories of a similar orientation. These categories were then examined to identifying patterns and points of connection, which led to the development of subordinate themes. Subordinate themes were defined by the reviewers collaboratively and then categorized with related emergent themes. This process helped to ensure the subordinate themes were reflective of participants’ descriptions of their experiences and the reviewers’ interpretation and understanding of their experiences. These themes and subordinate themes were reviewed together by both reviewers. When discrepancies emerged, the reviewers returned to session transcripts and/or discussed their understanding of the theme until they reached mutually agreeable decision. Additionally, study participants reviewed the themes to provide feedback, to ensure the themes and subordinate themes were reflective of their music and imagery experiences. This analytic process is designed to hold close to the experiences of the participants and maintain balance and integrate the reviewers’ understanding of their experience (Braun and Clarke, 2023a, 2023b).

Following the completion of the thematic analysis, phase two included an intertextual analysis. Intertextual analysis is a humanistic and conceptual framework that places the emphasis on understanding and viewing the text with the individual at the center and explores links on different interpretive levels (complexity, ideas, etc.) and considers the macro context of the narrative (Eliezer and Peled, 2023). This process recognizes that the text does not and cannot stand alone as a separate object, rather it is embedded in the context of the client (Eliezer and Peled, 2023; Elkad-Lehman and Greenfeld, 2011; Moseholm and Fetters, 2017). The themes and additional text were explored in the context of understanding the arc of the therapeutic process. This included examining the nine themes, and how they indicate and represent participants’ therapeutic process. Transcript excerpts that represented the nine themes were re-examined and entire transcripts were reviewed again to identify additional excerpts that may be relevant to the intertextual analysis.

## 4 Results

Over the 12-month time frame of the study, 6 sessions were missed, and 8 sessions had to be rescheduled, resulting in 122 sessions, of which 116 were facilitated. Analysis of session transcripts resulted in nine imagery subthemes with three main emergent themes: emotional landscape, relationships, and transformation and growth. Table 2 includes the subthemes of emotional landscape which include: feeling stuck, acknowledging emotions, and working through unresolved emotions. The subthemes indicate participants’ process in navigating aspects of their emotions from feeling stuck in their life to beginning to connect with and learning to express and regulate their emotions, as well as working through difficult and unresolved emotions.

**Table 2:** Subthemes of emotional landscape.

Feeling powerless and stuck	Feeling powerless
	Not possessing the strength or ability to move forward in one’s life
	Feeling stuck or trapped
Acknowledging emotions	Identifying emotions
	Claiming emotions
	Allowing self to begin to feel difficult emotions
Working through unresolved emotions	Practice tolerating difficult emotions
	Exploring unresolved emotions
	Working through emotions related to trauma

The next theme that emerged focused on relationships. Table 3 describes the various relationships represented in participants’ music and imagery sessions including self, others, and the eating disorder. In each of these subordinate themes, participants explored various aspects of each of these relationships including exploring and working through related challenges. Participants’ imagery also focused on the intersection of these relationships, such as experiencing the impact of their eating disorder on their relationship with self and others.

**Table 3:** Subthemes of relationships.

Self	Exploring relationship with body
	Discovering dissonance in feelings toward self
	Working to find self-acceptance
Others	Experiencing disconnection from others
	Discovering the need to connect with others
	Exploring how to connect with others
Eating disorder	Experiencing personification of eating disorder
	Discovering the negative and destructive nature of eating disorder
	Separating from eating disorder

The subthemes of transformation and growth are explicated in Table 4 and include finding strength, change, and empowerment. These represent their process of discovering their strengths as they faced fears, finding a desire to change and to live differently, engaging in a healing process, and building inner resources and self-confidence. These subthemes indicate finding the strength and capacity to confront their fears, make a conscious choice to change, and experience a sense of transformation and growth as a result.

**Table 4:** Subthemes of transformation and growth.

Finding strength	Facing fears
	Developing insights
	Experiencing physical and emotional strength
Healing images	Recognizing desire to change
	Experience of letting go
	Engaging images that foster healing
Empowerment	Discovering inner resources
	Developing belief in self
	Gaining confidence in abilities

The intertextual analysis of imagery themes illustrates an arc of participants’ therapeutic process. This arc begins with identifying one’s current circumstance of feeling stuck, then the need to work through difficult and unresolved emotions, which transitions to exploring and addressing relationships (self, others, and ED), and then discovering and integrating insights that foster change, growth, transformation, and sense of empowerment. In the process of reviewing these subthemes, what emerged is an arc of a process reminiscent of the *Hero’s Journey* (Campbell, 1993). These emergent themes were reviewed and compared to the stages of the *Hero’s Journey*. Table 5 explicates the emergent themes relative to the *Hero’s Journey* stages, and imagery excerpts that illustrate each. Comments and questions in parentheses in the imagery theme excerpts are statements or questions from the MT-BC during the music and imagery session. These statements and questions are focused on supporting the participants’ music and imagery experience to deepen their exploration, working to engage other senses, or offering encouragement and support.

**Table 5:** Hero’s journey imagery.

Imagery theme	Stage of hero’s journey	Imagery theme excerpts from transcripts
Unresolved	Ordinary world	“I am holding a white box with a green felted bow. It is from my emotions mother’s shop. I do not know what is inside, but I feel sad and angry that she left me this ‘gift’ to deal with. I feel sick as I hold it. I think it holds all the secrets that my family buried, and I am left holding onto them. (How do you feel as you hold them?) It is too much responsibility to hold onto them and I feel sick. I run to the lake and throw up in it.”
		“I am holding the dog leash, and the dog is pulling me. I am pissed. No one understands I was raped. What happened to me was forgotten due to the drama of my parents’ divorce. I am angry that no one figured it out. I am so good at covering things up. They should have figured it out. There was too much going on for them to pay attention. I was invisible.” (How does that feel?). I was just a kid, stuck in the middle, and taking care of myself.” (Just let the tears come).
Powerlessness and stuck look	Call to action	“I am at his house. I feel stuck, glued to the wall. I cannot get away. (How do you feel?) Trapped. He is sitting there with a menacing on his ghostly face. I feel sick, like I need to throw up. I feel so stuck.”
		“I am holding a ceramic bowl. It is heavy.” (What do you notice?) “There is beautiful blue water in it. There are tears falling in it. (Your tears?) “They feel like my tears, but I’m not crying. I feel really sad.” (Where do you feel it?) “In my eyes and chest. I feel it in my stomach. I feel so stuck, it hurts, I cannot move. No one can hear me. No one can hear me. I am all alone. I am broken and I cannot be fixed.”
Challenge of facing fears	Refusal of the call	“I am curled up behind the door. |I feel the cold from the stone floor on my legs. (How do you feel?) I am scared. I feel the fear throughout my body, my muscles are tense, and I feel frozen. I am unable to move. (What do you see?) I see light shining from underneath the door. I want to reach out and touch it, but I am too afraid. I am frozen in my fear.”
		“I am stuck underneath a large boulder. I can feel it pressing down on my body keeping me trapped and unable to move. (What do you notice about the boulder?) It is my ED, and it uses my fears to make the boulder bigger and heavier. (How are you feeling?) I feel weak and I am afraid I will never be able to break free.”
Relationships (self & others)	Meeting the mentor and allies	“I am at the bottom of a big, black pit. My feet are too slippery to be able to move.” (What are you feeling?) “A sense of urgency. I want to scratch and press against my legs, the pressure and compression feels grounding. When I feel that pressure I can tolerate being in my body.”
		“I am at the base at of a cavern pit. My feet are too heavy to move, and my arms are too weak to reach up. My neck is too weak to look up. I hear voices around me screaming, ‘do not look up, never look up. No one is coming to help you’. (How do you feel?) “I feel weaker and weaker as I hear this. I hear a tiny voice within me say, ‘look up, trust and look up’. (Can you look up?) “I slowly lift my head and as I begin to see glimpses of light, I see the arms of my loved ones reaching down to me. They are there. They want to help. They tell me they love me. (How does that feel?) “It gives me strength to reach out and grab their arms. (They are there). They were there all the time; I was just too afraid to look up.
ED personified	Facing the enemy and crossing the Threshold	“I see the wounded little girl and the ED is criticizing and harassing her. I watch as she is overpowered by it and scared. She is not what ED says she is. She is not bad; she is a little girl.” (How do you feel?) “I feel sad and angry. I have said those same negative things to the little girl. She is not the monster that the ED describes her as.”
		“I am looking in a mirror. I see a face emerging. It is the face of the Joker, with black and white on its face. It looks violent and scary. He puts tape over my mouth and begins yelling at me and telling me what to do. He is the ED. He tells me I screwed up, that I am bad and that is why someone abused me. (What do you want to do?) I take the tape of my mouth and tell him, that is why I threw up everything up, so I could shrink away. He tells me I have to keep doing that to make my body perfect and to make it clean. He promises if I keep doing this it will make my life better.”
Addressing trauma & unresolved pain	The approach,	“I do not know how to heal those wounds. There are layers of hurt and the ordeal all over me. They suffocate me.” (Where do you feel it?) In my throat and chest. I do not want to feel it I do not want to be touched down there. (Let the tears come.) When my grandma died everything fell apart.” (Breathe and be with the music). “She was not there to protect me anymore and I could not protect myself.”
		“I am in my aunt’s garden. I loved her, but her garden is dead now. As I look around, I hear the words, ‘you cannot recreate her garden, you need to create your own’. I realize it is time to dig up the garden because it died for a reason. (What are you feeling?) I feel angry as I dig. I need to stop listening to my mom, the ED, and my self-doubt. I feel the hurt as I dig in the soil. I need to grow what I want to grow in the garden and within me. “
Healing images	The reward	“I see the ocean and it is a beautiful blue color.” (How do you feel as you see it?) I feel connected to it. I feel the sun shining down on me as I step into the water. I am swimming in the ocean. (How does that feel?) Wonderful! (What do you notice?) The water is clear, and I can see everything. I feel safe and free. The water is cleansing. I feel it washing away the hurt and pain. This is sacred place to honor and respect.”
		“I am in my grandparent’s backyard. I see the lily of the valley and the purple violets. (What else do you notice?) I can smell the lily of the valley. I see the blue sky. I feel safe here. (Do you feel that in your body?) I feel it all over, a general sense of well-being and contentment. (Take that in.) I can breathe easily and I begin to sing. I feel the strength of my voice and how the air in my lungs support it. I notice how my body moves to support my breathing and singing. I do not judge it; I appreciate it and love it for what it is able to do.”
Empowerment	The road back, the resurrection, return with the elixir	“Lightning is all around me. I do not want to be a target or get hit. (What can you do?) “I can defend myself. I can actually catch the lightning bolt; it becomes powerful on my side. (Powerful) I do not know what do to with it yet. I feel like Wonder Woman. I can harness this power and learn how to use it. It’s possible to have it become a part of me and to use it for good like Wonder Woman, Xenia” (Strong women).
		“There is a large bowl of lemons on the counter and several laying on the table. I am struck by the bright and vibrant color of the lemons. I grab the lemons on the table and place them in the large bowl. The bright yellow color of this bowl of lemons is energizing. (Do you feel that energy?) I feel it moving through my hands and arms and into my chest. (How does that feel?) I feel a strength and a sense of power within. It is a strength I have never felt before. (Let yourself take that in.) I feel this strength radiating through my whole body.

## 5 Discussion

The aim of this secondary analysis was to understand participant’s experiences of GIM through a thematic analysis of their imagery and exploring their overall therapeutic process through intertextual analysis. Research to date on the use of GIM with individuals in ED treatment is limited to clinical case studies and a descriptive feasibility study that examined participants perceived benefits and challenges (Heiderscheit, 2015a, 2015b, 2023; Trondalen, 2016a). Comparatively, research on the use of GIM with other client populations has explored how the music supports the imagery experience, fosters different types of imagery, and has examined imagery themes that emerged from a brief series of GIM sessions (Beck, 2015, 2019; Heiderscheit, 2017, 2022; McKinney and Honig, 2017; Honig et al., 2021; Short, 2021; Testa et al., 2020). While this study strives to understand individual’s experiences of GIM, it is limited in scope by the inclusion of eight participants engaged in ED treatment. Therefore, the results are limited as to how they may be generalized more broadly to clients in ED treatment.

### 5.1 Addressing complexity: a neuroaesthetic process

Participants in this study had complex profiles due to their ED diagnosis, impact of physiological and neurological ED symptoms, multiple co-morbid mental health diagnoses, physical impact of ED trauma, and numerous previous treatment episodes. The imagery themes that emerged indicate that as participants engaged in this series of embodied aesthetic experiences, that fostered a complex interplay allowing them to project their unique and subjective thoughts, feelings, and memories onto the music and imagery. Their engagement in GIM sessions fostered exploration and externalization of their inner experiences they previously struggled to make meaning of and comprehend. In the context of this supportive and aesthetic psychotherapeutic process participants engaged in tolerating, exploring, learning to manage/regulate, and work through difficult emotions that they had avoided or repressed. Participants embodied engagement with their emotions and memories in image form fostered deeper exploration through various sensory modalities integrating interoception, proprioception, and exteroception signals (Vaisvaser et al., 2024).

Through their GIM sessions, participants were able bring these repressed emotions and memories from the subconscious into their conscious awareness, framing them in image form, allowing them to exert control over how long and the ways they engaged with them. This externalization process helped them build and develop their emotional capacity and agility, as well as foster their motivation to address other therapeutic issues and explore other aspects of their life (David, 2017; Patching and Lawler, 2009; Vaisvaser et al., 2024). Building their emotional regulation skills facilitated exploration of intrapersonal and interpersonal relationships. Exploring relationships included experiencing their ED in symbolic form which provided opportunities to distance themselves from their ED, begin to see its destructive nature, and gain awareness and insight into the negative impact of the ED on their body, health, and interpersonal relationships. This symbolization and metaphoric representation of the ED engaged cognitive and emotional processes that served as a pivotal turning point in helping participants understand the severity and impact of their ED (D’Abundo and Chally, 2004; de Witte et al., 2021; Gargaro et al., 2016; Heiderscheit, 2017). Addressing emotionally challenging and underlying issues was a key element in supporting participants’ process of change. This suggests that while an ED is a collection of symptomatology, there are underlying issues impact these symptoms and that need to be addressed (Cockell et al., 2004; Heiderscheit and Madson, 2015; LaMarre and Rice, 2021; Patching and Lawler, 2009; Strober, 2010; Wonderlich et al., 2012). In the GIM sessions participants were able to address underlying issues that helped facilitate transformation and growth. As participants explored images as they unfolded, it helped them reframe and recognize that they could face their fears, discover their strengths, and find their inner resources. The embodied nature of this experiential work in GIM fostered healing images that served as the impetus of change and supported participants decision to work on letting go of the ED. The series of GIM sessions helped participants discover and develop a desire to live their life differently and to see themselves in a new way (Gargaro et al., 2016).

Addressing unresolved and underlying emotions also included working through feelings, memories, and experiences related to childhood and past trauma. A participant shared, “I was so afraid to approach working through my trauma. I found in the GIM sessions that I did not approach my trauma work until I felt I had the resources to do so. It was still uncomfortable, painful, and difficult, and I was able to work through it at my own pace.” (Heiderscheit, 2023, p. 6). The externalization process provided participants the psychological distance to process their trauma in their own way and at their own pace. Additionally, the symbolic and metaphoric nature of the music imagery allowed participants to explore their inner experiences and making meaning of them through engagement with embodied cognitive and affective processes (Vaisvaser et al., 2024). Another participant reported, “I was having a difficult time dealing with the trauma. EMDR, CBT & DBT were somewhat helpful, but I kept getting stuck in the trauma again and again. GIM helped me work through the trauma in my own way, my own time and in a way that allowed me to feel safe.” (Heiderscheit, 2023, p. 6). This indicates their ability to exert psychological control and pace their process in their GIM experiences, fostered their safety throughout the process. Stimulating and engaging the perceptual, cognitive and affective brain functions were key in beginning to explore behavioral and psychological change.

### 5.2 Process of change and transformation

The intertextual analysis of imagery themes represent a therapeutic arc that indicative of a process of change that is evident in the *Hero’s Journey*, which is a metaphoric story about a traveler facing challenges on their adventure, which tests, pushes and requires them to learn new skills to emerge from the experience transformed, and empowered to bring what they have discovered into their life (Campbell, 1993; Williams, 2016, 2017). The *Hero’s Journey* is utilized to create an arc in a story, and it is also representative of transformation and change which is reminiscent of the therapeutic process (Campbell, 1993; Vogler, 2020).

When the nine imagery themes that emerged from the thematic analysis were analyzed in the context of participants therapeutic process (intertextual analysis), it became evident that they follow the arc of the stages of the *Hero’s Journey*. Evidence of this mythic journey being represented in their series of GIM sessions, suggests participants engagement with a shred symbolism and metaphor across the arc of their therapeutic process, as well as the engagement cognitive and affective processes that supports a client’s transformation (Coutinho Cortes et al., 2024; Vaisvaser et al., 2024). Transformation in the therapeutic process aligns with the *Hero’s Journey* in which the hero is transformed after overcoming challenges through determination of developing awareness, new insights, and skills (Williams, 2016, 2017). The findings from this intertextual analysis and metaphoric representation of the *Hero’s Journey* has appeared in the imagery in the GIM process with patients facing other mental health and medical crises (Heiderscheit, 2022; Short et al., 2011). The representation of *Hero’s Journey* across psychological and physical health journeys, indicates both present struggles and challenges for an individual to work through to experience transformation and change.

### 5.3 Implications of the study

These findings along with previous research indicate that whether an individual is facing physical health crises, mental health issues, or a combination, there is a similar process of self-exploration and discovery that is key to foster healing and recovery as a part of one’s health journey (Heiderscheit et al., 2022). Given EDs include psychological and physical health issues, GIM offers a wholistic psychotherapeutic approach treatment to address individual’s complex needs. Engaging individuals diagnosed with an ED an aesthetic, supportive psychotherapy process is key to accessing perceptual, cognitive, and affective brain functions which are vital to their treatment and recovery (Vaisvaser et al., 2024). Further, the process of discovering and reclaiming their inner resources allowed participants to gain a sense of mastery and self-efficacy which helped them to build a sense of internal safety and empowerment to make healthy choices and decisions for their life through a short series of between 11 and 17 GIM sessions (Heiderscheit, 2023; Malchiodi, 2018; Ogden et al., 2006; Short et al., 2011; van der Kolk, 2014).

## 6 Conclusion

EDs are complex and have multiple factors that influence their development, as well as a myriad of mental and physical comorbidities that impact and complicate the treatment and recovery process. ED treatment needs to integrate a wholistic approach that is tailored to the ED symptomology, the unique context of the individual, and holds the capacity to access the subconscious to address underlying issues. A focus on engaging clients in identifying and working through these issues in experiential and embodied ways integrates neurobiological and psychological process that fosters meaning making, mastery, and empowerment (Kaufman, 2022; Coutinho Cortes et al., 2024; van der Kolk, 2014; Vaisvaser et al., 2024). While empirically based and manualized treatment ED approaches have been most researched, the body of evidence exploring and examining the use of CATs in ED treatment is growing (Agras and Robinson, 2018; Testa et al., 2020; Heiderscheit, 2023). Further exploration of embodied aesthetic approaches is needed to expand the neuroscientific understanding of the process with individuals in ED treatment.

Valuing and integrating the lived experiences and wisdom of client’s, provides the opportunity to inform and tailor experiential methods and approaches for ED treatment (Guarda et al., 2018; Lotter and van Staden, 2022; Silber et al., 2011). Further, understanding client’s experiences in GIM provides insight into areas of therapeutic change which helps to inform the choice of outcome measures for future research. Additionally, recommendations for future research include the integration mobile brain imaging (MoBI) data to gain insight into neurodynamic engagement and therapeutic interactions across a series of sessions. Future research could also focus on implementing outcome-based research targeted and addressing the unique needs of different ED diagnoses. Indepth aesthetic and experiential methods like GIM engage clients in a therapeutic process that fosters their discovery, paced to meet their unique needs, and engaging them in meaning making experiences that support their treatment and recovery. Finally, the body of existing evidence can be used to inform implementing more rigorous research exploring the use of the CATs with clients in ED treatment.

The high economic costs of ED treatment and disease burden of EDs indicate an urgency to reduce their impact and identify effective treatment protocols (Streatfeild et al., 2021). ED treatment programs integrate CATS and experiential modalities into their regular programming to provide clients and their families with an integrative approach to treatment and recovery. Individuals trained and credentialed in the Bonny Method of Guided Imagery and Music (GIM) can provide individual or group sessions to support clients in addressing the issues underlying the ED and discovering and developing strengths needed for recovery. A holistic approach to ED treatment can address the complex needs of clients and may hold the potential to reduce recidivism and decrease overall costs of treatment.

## Data Availability

The datasets presented in this article are not readily available because datasets include full session guided imagery and music transcripts. Clients did not consent for full session transcripts to be shared. Requests to access the datasets should be directed to Annie.Heiderscheit@aru.ac.uk.
